# Coexistence of Riedel’s Lobe and Supernumerary Kidney as Random Imaging Findings

**DOI:** 10.7759/cureus.27191

**Published:** 2022-07-24

**Authors:** Kyriaki Georgiadi, Vasileios Balomenos, Gregory Tsoucalas, Aliki Fiska

**Affiliations:** 1 Department of Anatomy, Democritus University of Thrace, Alexandroupolis, GRC; 2 Department of Radiology and Interventional Radiology, University Hospital of Alexandroupolis, Alexandroupolis, GRC

**Keywords:** renal developmental anomaly, liver, abdominal surgery, anatomical variation, radiology

## Abstract

Supernumerary kidney (SNK) is a rare congenital anatomical variation usually detected incidentally via imaging. Although a random finding, it may present with hydronephrosis, calculi or malignancy. Both its vascularization and its drainage vary significantly, depending on its location and shape. Riedel’s lobe is a normal, though rare, variant of liver anatomy presenting either as a downward projection of the inferior border of the right liver lobe or as a triangular pyramidoid projection to the right of the gallbladder.

We present a case of a 71-year-old man who was initially admitted to the hospital for backache. Computed tomography (CT) imaging revealed the simultaneous occurrence of a left supernumerary kidney and Riedel’s lobe. The SNK lay caudally to the normal kidney, it was supplied by a branch of the superior mesenteric artery and its drainage was supported by a bifid ureter. The Riedel’s lobe represented the “tongue-like” variant without causing any symptoms to the patient.

Both entities should be monitored carefully, as their presence may require surgical management should they raise a sequence of symptoms or, as in this case, modify the surgical plans in the context of other coexisting medical events.

## Introduction

A supernumerary kidney (SΝK) is an encapsulated additional parenchymal organ, supported by its individual vasculature and collecting system. It is considered a very rare congenital anatomical variation with only a few cases reported worldwide [1,2]. The diagnosis of supernumerary kidney is established when the total number of kidneys exceeds two, regardless of the coexisting variations that may occur (e.g. horseshoe). From an embryological perspective, supernumerary kidneys are thought to be formed by an abnormal formation of two metanephric blastemas within the nephrogenic cord connected with partially or completely duplicated ureteral buds, but its origin remains unclear [3].

The type of SNK drainage by an individual ureter or a bifid ureter can distinguish two separate entities of this condition. The location of the supernumerary kidney in relation to the ipsilateral kidney usually follows the type of its drainage: when it is located cranially, an individual ureter is present, and when caudally, which is more common, it is drained by a bifid ureter. Blood supply also varies depending mostly on the position of the additional kidney compared to the major one [3,4]. SNK is generally characterized by its smaller size compared to the normal kidney, its appearance (e.g. horseshoe formation) and its poor functionality, as it can be compromised by vascularization.

A Riedel’s lobe is an anatomical variation of the liver, appearing as a downward tongue-like projection of the inferior border of the right lobe or as a triangular pyramidoid projection to the right of the gallbladder [5]. It is usually identified as a palpable mass at the right hypochondrium, and it is mostly asymptomatic. Nevertheless, Riedel’s lobe is included in the differential diagnosis of the right abdominal masses, as it is involved in hepatic malignancies [6-9].

This report presents a unique case of a 71-years-old male with a supernumerary kidney on the left side, as well as a Riedel’s hepatic lobe.

## Case presentation

A 71-year-old male was admitted to the University Hospital of Alexandroupolis complaining about a severe backache. Anti-inflammatory drugs were administered to him and he was discharged. A month later, the recurrence of the symptoms led to his admission for further clinical investigation. Laboratory tests revealed anemia and acute renal failure which was treated conservatively. Anemia was thoroughly investigated for its origin and a sigmoid colon mass was detected via imaging.

Pre-surgery testing revealed an aneurysm of a jejunal branch of the superior mesenteric artery. A Riedel’s lobe of the “tongue-like” variant, as well as a supernumerary left kidney, were also detected, as random imaging findings. 

**Figure 1:**
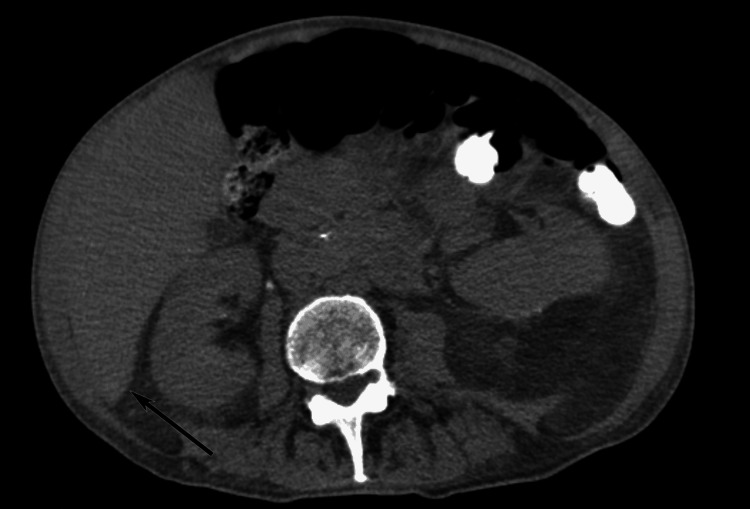
Riedel's Lobe Abdominal computed tomography (CT) scan without intravenous contrast medium. The Riedel’s lobe appearing as a tongue-like projection of the right lobe’s inferior border of the liver is highlighted (black arrow).

**Figure 2:**
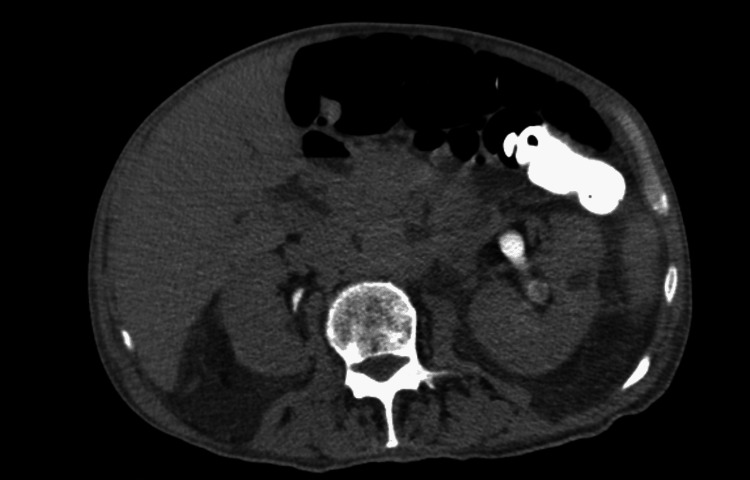
Kidneys Abdominal computed tomography (CT) scan without intravenous contrast medium. Normal kidneys.

**Figure 3:**
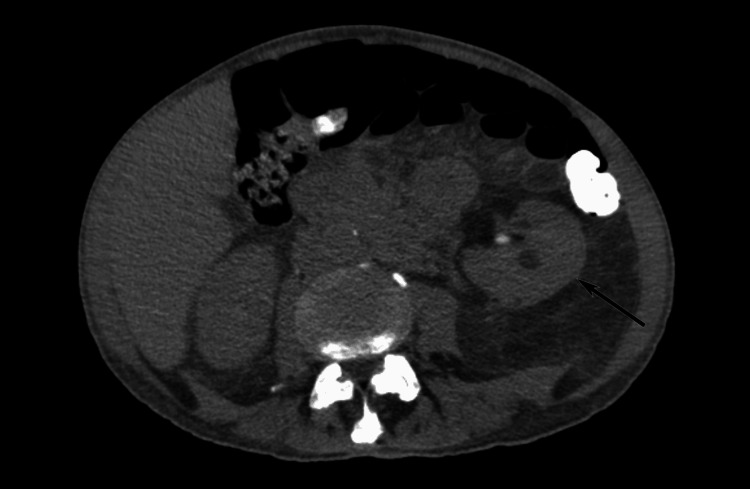
Supernumerary kidney Abdominal computed tomography (CT) scan without intravenous contrast medium. The supernumerary kidney is located on the left, caudally to the left normal kidney (black arrow).

The SNK was fully separated from the normal kidney and located caudally to the latter. Its drainage was supported by a bifid ureter and its vascularization was supplied by a separate artery derived from the superior mesenteric artery (Figures 1-3).

**Figure 4:**
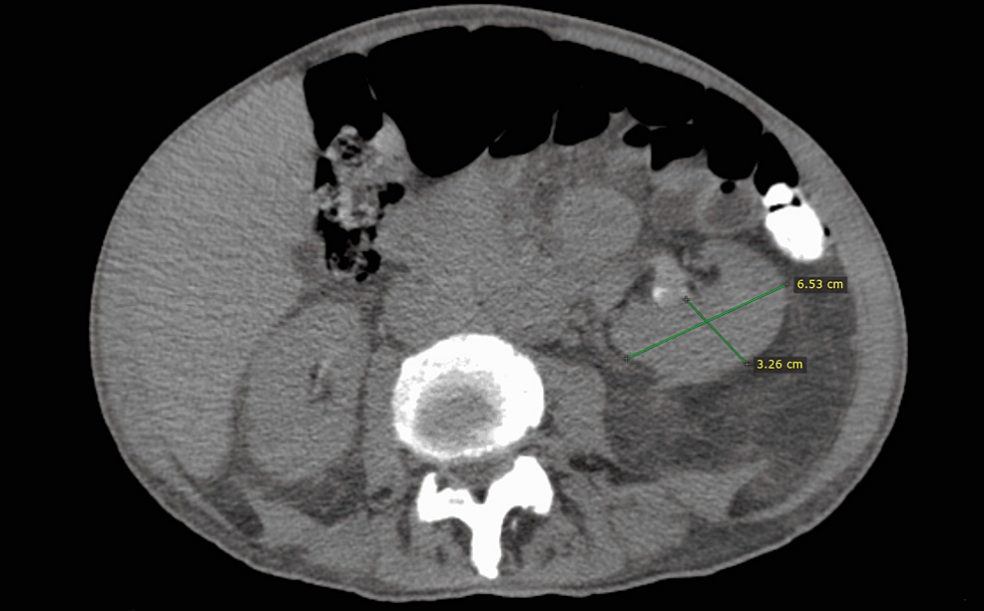
Supernumerary kidney's dimensions Abdominal computed tomography (CT) scan without intravenous contrast medium. The dimensions of the supernumerary kidney are highlighted.

The SNK’s dimensions were 6.53 x 3.26 cm, significantly smaller than the normal kidney that usually ranges between 10 and 14 cm long in males (Figure 4).

Sigmoidectomy was the preferred choice of surgical treatment, in combination with simultaneous jejunal arterial aneurysm resection. Postoperatively, the patient presented symptoms and radiologic signs of intestinal obstruction (ileus), which required an ileostomy procedure. The postoperative course of the patient remained eventful as renal failure caused severe electrolytic disorders. Two months post-surgery, the patient died from septic shock due to a failed intestinal anastomosis and intraabdominal contents leakage.

## Discussion

We report a unique case of a 71-year-old male with a supernumerary kidney on the left side concurrent with a Riedel’s liver lobe.

SNK is an uncommon anomaly with less than 100 cases reported to date. It can be associated with several congenital abnormalities such as ectopic ureteric opening, ureteral and vaginal atresia, among others. When this is the case, this cluster of rare diseases further complicates the standardization of a diagnosis protocol. Still, SNK is considered mostly a random imaging finding [10,11].

According to the available data, either side presents equal frequency in relation to the supernumerary kidney’s location. In our case, the SNK was located on the left side without the “horseshoe” malformation, following the typical pattern. The drainage system of the SNK varies significantly from an independent to a bifid ureter with the latter being the most anticipated type of drainage and appearing in our case. The SNK typically depends for its arterial supply on its own separate artery, albeit anatomical vascular variations may occur. Once again, the SNK under study abides by the rule of a separate artery.

Although SNK is mostly asymptomatic, it can be accompanied by a number of symptoms, such as hypertension, abdominal discomfort, hydronephrosis, or present as a palpable mass. Even though none of the above-mentioned clinical signs, except for a complaint of backache, were present in our case, the patient was diagnosed with acute renal failure soon after his second admission to the hospital. Nevertheless, the clinical findings of our patient’s several comorbidities suggest that neither back pain nor acute renal failure can be directly associated with the SNK.

Riedel’s lobe is an uncommonly encountered morphological variation of the hepatic lobulation, where the inferior border of liver segments 5 and 6 projects inferiorly to the right of the gallbladder [9]. It presents an incidence which varies from 3% to 15% [10,11]. The projection tends to elongate towards the lower pole of the right kidney, shaped either as a “tongue-like” or as a triangular pyramid. In this case, Riedel’s lobe was acknowledged as a “tongue-like” variant. Although this accessory liver lobe is usually accompanied by a series of minor clinical symptoms, such as nausea, abdominal discomfort, and constipation [12], the patient in our case did not manifest any of them. Riedel’s lobe, being nothing but a normal variant, presents with an excellent prognosis and it is usually an incidental finding on an abdominal computed tomography (CT) scan done for an unrelated reason.

To our knowledge, this duet of anatomical variants depicts a sui generis case that has not been previously reported. The left location of the SNK, contralateral to Riedel’s lobe, invalidates any attempt to form hypotheses about an embryological connection of their coexistence. Furthermore, taking into consideration that Riedel’s lobe is more frequently encountered in females, with a male to female ratio of 1:3, the case we present exhibits even greater peculiarity [10,11,13].

Both anatomical entities require careful management to ensure an optimal outcome for the patient. SNK management depends on renal function, patient’s symptoms, and related complications. Although asymptomatic or minimally symptomatic cases are favored for a scheduled follow-up to detect early complications, surgical intervention (nephrectomy) is considered the treatment of choice for SNKs with associated comorbidities. It is important to highlight that scientific data suggest that variations in renal vascular anatomy such as multiple renal arteries that inevitably accompany an SNK, or renal parenchymal congenital anomalies, may affect aortic endograft procedures [14]. Prior consultation is therefore recommended for patients who might undergo surgery for aneurysm repair, as a supernumerary renal artery should be taken into consideration [14].

Regarding Riedel’s lobe, it is a mostly asymptomatic accessory liver lobulation that usually requires no special treatment than a regular follow-up and monitoring [15]. Surgery, resection or laparoscopy [9,16] is considered only in cases of complicated Riedel’s lobe involving hydatid cysts, metastatic lesions or liver hypertrophy. Furthermore, there are some studies that report an association between this accessory liver lobe and pedunculated hepatocellular tumors with an incidence of 0.2-4.4% [17,18]. In addition, Riedel’s lobe has been also related to both simulation of acute appendicitis and gastric outlet obstruction by Iskra et al. [19] and Akbulut et al. [20], respectively.

## Conclusions

Random cases enrich physicians’ perspectives. A third kidney with an abnormal Riedel’s lobe is a rare medical event that may raise diagnostic issues and create surgical complications during abdominal interventions. Modern imaging techniques (CT) with advanced protocols play a vital role in assisting physicians' choice of treatment in a more personalized approach towards the patient. Although both entities are rarely linked with major complications, it is important to be attentive, especially in cases where the surgical approach is highly considered. Another interesting point that needs to be brought forward is the embryological descent of these cases, an approach mostly neglected as physicians focus on treating symptoms.

In general, anatomic aberrations, even those that don’t normally disrupt the body’s function, could accompany, induce, or perplex clinical signs and should always be kept in mind. Case reports contribute to this pathway enhancing our knowledge of anatomic variations.
